# An abrupt rise of coagulation error messages on ACL TOP automated analysers

**DOI:** 10.11613/BM.2019.021002

**Published:** 2019-06-15

**Authors:** Bas Calcoen, Koen Desmet, Pieter Vermeersch

**Affiliations:** 1 Department of Laboratory Medicine, UZ Leuven, Leuven, Belgium; 2 Department of Cardiovascular Sciences, University of Leuven, Leuven, Belgium

**Keywords:** preanalytical phase, blood coagulation test, diagnostic error, blood specimen collection

## Abstract

**Introduction:**

Blood coagulation tests (BCT) are very important for clinicians to diagnose bleeding or thrombotic disorders and to monitor anticoagulant therapy.

**Case description:**

On a Saturday morning, a laboratory technician noted an abrupt rise in the number of coagulation error messages on our ALC TOP analysers. Visual inspection revealed the presence of partially and/or fully clotted citrate tubes and prompted the clinical biologist to further investigate a potential preanalytical cause.

**Considered causes:**

Partially or fully clotted blood in citrate tubes can have multiple causes including improper mixing of the tube, under- or overfilling or combining blood samples from different tubes into one citrate tube.

**What happened:**

The affected citrate tubes originated mostly from the same clinical departments. Moreover, all the affected tubes shared the same lot number (1 of 7 in use at the time). Visual inspection of 7 unopened boxes of 100 citrate tubes of this lot number revealed one box with nine completely empty and two partially filled tubes and one box with two partially filled tubes. No under-filled tubes were found in the other 5 boxes.

**Discussion:**

The blood to additive ratio is crucial for BCT. A sudden rise in clot errors should trigger a thorough investigation to identify the cause.

**Main lesson:**

Laboratories should regularly monitor and evaluate the percentage of clotted samples as a quality indicator at scheduled time points. A problem with the volume of additive in citrate tubes should be considered as a possible cause.

## Introduction

Prothrombin time (PT) and activated partial thromboplastin time (aPTT) are important for clinicians to diagnose a possible bleeding and/or thrombotic disease (*1*–*3*). Additionally, these blood coagulation tests (BCT) are frequently used for monitoring anticoagulant therapy. Prothrombin time, defined as an international normalized ratio (INR), and aPTT are used for monitoring vitamin K antagonists and unfractionated heparin, respectively (*1*, *2*, *4*). Clinical laboratories must therefore guarantee an accurate determination of BCT since unreliable results could jeopardize patient safety and medical decision-making (*1*, *5*). It has been stated that approximately 70% of laboratory errors occur in the preanalytical phase (*2*, *5*, *6*). The mandatory quality indicators for the total testing process proposed by the International Federation of Clinical Chemistry and Laboratory Medicine (IFCC) working group Laboratory Errors and Patient Safety include the “percentage of number of samples clotted/total number of samples with an anticoagulant” and the “percentage of number of samples with inappropriate sample-anticoagulant volume ratio/total number of samples with anticoagulant” (*7*). Clinical laboratories need to be suspicious for potential preanalytical errors when there are unexplained abnormal BCT values and provide adequate and fast solutions to minimize the potential safety risk of the patient (*2*, *5*, *6*).

## Case description

**Figure 1:**
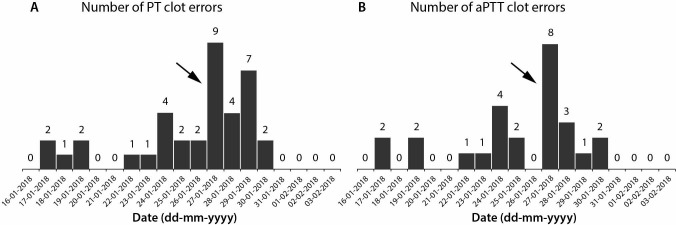
Timeline of the number of clot errors *per* day for PT (panel A) and aPTT (panel B). The problem was noticed on Saturday morning 27^th^ of January (black arrow). PT - prothrombin time. aPTT - activated partial thromboplastin time.

On a Saturday morning, one of the laboratory technicians noted a sudden rise of the number of ‘coagulation error’ triggers on our two track-coupled automated ACL TOP 700 analysers (Instrumentation Laboratory Company, Bedford, USA). Visual inspection of the affected citrate tubes (2.7 mL BD Vacutainer plastic whole blood tubes containing 0.109 mmol/L (3.2%) buffered sodium citrate, Milan, Italy, ref. no: 363048) by the technician revealed that several samples were completely clotted. In other tubes, there was no visible clotting, but several small clots were detected by inserting 2 wooden applicator sticks into the sample (*6*). After reporting these findings to the supervising clinical biologist, a query of recent BCT results was executed which revealed a rise of aberrant PT and aPTT values in the last few days (see Figure 1A and 1B). To minimize the potential impact of incorrect BCT results, immediate actions were taken to identify the cause.

## Considered causes

Coagulation tubes (light blue tops) are almost exclusively used for BCT (*1*–*3*, *5*, *8*). Citrate reversibly binds and removes calcium that serves as an essential co-factor within the coagulation cascade, and consequently halts the clotting process (*9*). Several preanalytical elements may provoke partially and/or fully clotted blood in citrate tubes. Possible explanations are 1) prolonged venous stasis, 2) a difficult phlebotomy which causes ‘in vitro’ haemolysis, 3) use of 23 or 25 Gauge (G) needles, 4) leaving blood in a syringe too long when there was no Vacutainer system used to perform a phlebotomy, 5) improper mixing and/or inverting of the tube, 6) under- or overfilling of the citrate tube or 7) combining blood samples from different tubes into one citrate tube, especially samples from serum tubes (contain a clot activator) (*2*, *5*, *6*, *10*, *11*).

Lippi *et al.* evaluated and validated a novel preanalytical module on an ACL TOP analyser. Their interference studies enabled reliable upper limits for sample rejection, namely 3.6 g/L, 233 µmol/L and 16.4 mmol/L, respectively for cell-free haemoglobin, total bilirubin and triglycerides. These thresholds were consistent with the manufacturer’s instructions for use (*8*). In addition, knowing how to interpret clotting reaction curve error messages on an ACL TOP analyser can enhance the appropriate laboratory follow-up for a failed sample result (*12*). According to the manufacturer’s package leaflet, coagulation errors can be caused by 1) low fibrinogen values, 2) improper reagent placement or 3) if no clot has formed before the end of the acquisition time (*13*).

## What happened?

**Figure 2:**
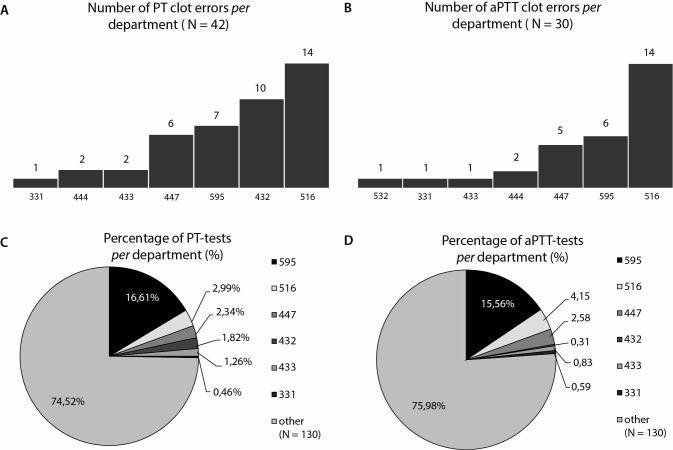
Number of clot errors *per* department for PT (panel A) and aPTT (panel B) and the relative percentage of PT and aPTT-test requests *per* department (panel C and D). PT - prothrombin time. aPTT - activated partial thromboplastin time. Department 595 is the emergency department. The other listed departments are the medical intensive care unit (516), abdominal surgery (447), cardiology, (432) bleeding disorders (433) and pediatric neurology (331).

As previously mentioned, the laboratory technician visually inspected the citrate tubes which had a ‘coagulation error’ trigger and found that multiple samples were completely solidified, while several small invisible clots were detected in other affected samples (*6*). After further inspecting these tubes, it became clear that most affected tubes originated from a few different clinical departments (see Figure 2A and 2B). The supervising clinical biologist suspected that there might be a problem with a certain lot of citrate tubes and removed the identification barcode labels that were put over the initial production label while performing the phlebotomy. At that time, seven different lot numbers of citrate tubes were in use in our hospital. Indeed, 44% of the most recent prolonged BCT results (52 of 118 results) were from samples drawn in tubes of one lot number, including 19 results which had a clot error. Fifteen of these 19 affected tubes were completely solidified, whereas the remaining four contained partially clotted blood.

**Figure 3:**
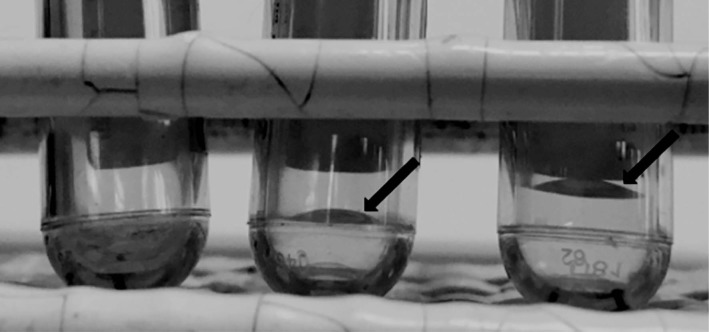
Visual control of tubes of the affected lot number revealed that some citrate tubes contained no (left) or only a limited amount (central) of trisodium citrate. The tube on the right contains an adequate amount of trisodium citrate. The black arrows indicate the filling level of trisodium citrate inside the tube.

Immediate action was taken by sending a warning message to the whole hospital to not use citrate tubes of the lot number in question and to send them back to the central logistic department. Subsequently, inspection of a number of unused tubes of the affected lot revealed that there were occasionally citrate tubes with no or only a limited amount of trisodium citrate inside the tube (see Figure 3). Additionally, we randomly controlled seven of the retrieved unopened boxes, each containing 100 citrate tubes of this lot. One box had nine tubes that were completely empty and two that were partially filled with trisodium citrate, while another box contained two citrate tubes with only a minimum amount of additive. No abnormalities were found in the other five boxes. It is very important that the standardized amount of trisodium citrate (105-109 mmol/L or 129 mmol/L) is respected to yield a correct blood to additive ratio of 9:1 (*2*, *3*, *5*, *6*, *14*). For this reason, all the test results derived from citrate tubes with the affected lot number could not be trusted.

## Discussion

It is known that unsuitable specimens still represent the leading source of nonconformities in the clinical laboratory, and especially for BCT determination (*2*, *6*, *8*). Continuous education and evaluation of respectively the phlebotomist and venipuncture procure is needed to minimize the preanalytical issues that could influence test results (*5*).

For haemostasis testing, a correct “order of draw” and grade of filling may be considered as the most important preanalytical factors (*1*, *2*, *5*, *6*). The Clinical and Laboratory Standards Institute (CLSI) recommends using a 3.2% citrate tube and a minimal citrate tube fill volume of 90% (*15*). Also, if winged blood collection systems are used for blood collection, a discard tube is needed to prevent under filling of the citrate tube because of the dead volume of these collection systems (CLSI guideline H3-A6). Ver Elst *et al*. validated the minimal tube fill volume and found that aPTT (minimum volume of 90%) was most sensitive to under-filling, whereas PT-INR and fibrinogen were relatively more insensitive (respectively minimum volumes of 73% and 63%) (*14*). In our laboratory, grade of filling is automatically controlled by our ACL TOP analysers and an alarm message is generated when the filling volume is below 70%. A filling grade of less than 90% can alter aPTT values in a statistically and/or even clinically significant manner (*5*, *14*). Therefore, we did validate and stated that for all BCT a cut-off filling grade of 70% can be used without retrieving a clinically significant impact on the results. None of the 118 controlled citrate tubes showed a significant under-filling. Although over-filling of the citrate tubes is not considered as a possible interference factor, the extreme low amounts of trisodium citrate in our situation led to an abnormal blood to additive ratio that was not enough to prevent coagulation. It must be mentioned that gently inverting the citrate tube approximately 4-5 times is important to maximize the anticoagulant effect of the trisodium citrate (*2*, *5*). It has been proposed in literature that inverting 8 to 10 times is needed to properly mix the blood sample with the additive, but the relative importance of this additional inverting manoeuvre has not been proven. However, vigorous mixing should be avoided (*2*, *5*, *6*, *11*). Of importance, the high relative proportion of BCT requests (see Figure 2C and 2D) by one of the departments (emergency medicine) that used tubes of the affected lot may have contributed to the rapid detection of this problem.

Nowadays, most modern coagulation analysers have built-in clot detection systems. For ACL TOP systems, every sample is pipetted into specific transparent wells, after which the coagulation cascade is started by adding the correct reagents. A coagulation curve based on turbidity measurement is constructed and using pattern recognition, a ‘coagulation error’ trigger is generated if the curve is abnormal. Samples with a deficiency of a certain clot factor or (partially) clotted samples both lead to an aberrant coagulation curve and therefore receive a coagulation error. In our case, the latter occurred and resulted in the detection of nineteen (partially) clotted samples drawn in citrate tubes of the affected lot.

At last, we contacted the manufacturer to warn that there was possibly a production problem of the citrate tubes. Because there is no individual control of the tubes, it is possible that occasional systematically under-filling because of a blocked or broken needle/pipe is missed. In all of the years that we worked with tubes delivered by this manufacturer, this is the first time that we encountered such an event. The manufacturer immediately issued a recall and no further problems were reported.

## Main lesson

In conclusion, the preanalytical phase is still the most import source for inconclusive results, especially for BCT. Not only a correct phlebotomy procedure and sample transport, but also a visual control of the tubes if they contain their additives and regularly monitoring the percentage of clotted samples as a quality indicator can help to minimize and/or prevent the clinical impact of a lot-dependent problem.

## References

[1] Bonar RA Lippi G Favaloro EJ Overview of hemostasis and thrombosis and contribution of laboratory testing to diagnosis and management of hemostasis and thrombosis disorders. Methods Mol Biol. 2017;1646:3–27. 10.1007/978-1-4939-7196-1_1 28804815

[2] Lippi G Favaloro EJ Preanalytical issues in hemostasis and thrombosis testing. Methods Mol Biol. 2017;1646:29–42. 10.1007/978-1-4939-7196-1_2 28804816

[3] Bennet ST, Lehman CM, Rodgers GM. Collection of Coagulation Specimens. In: Bennet ST, Lehman CM, Rodgers GM. Laboratory Hemostasis: A practical guide for pathologists. Second edt. Salt Lake City, USA: Springer International Publishing AG;2015.p.19-32. https://doi.org/10.1007/978-3-319-08924-9_2

[4] Bennet ST, Lehman CM, Rodgers GM. Monotoring of Anticoagulant Therapy. In: Bennet ST, Lehman CM, Rodgers GM. Laboratory Hemostasis: A practical guide for pathologists. Second edt. Salt Lake City, USA: Springer International Publishing AG;2015.p.135-72. https://doi.org/10.1007/978-3-319-08924-9_10

[5] Lippi G Salvagno G Montagnana M Lima-Oliveira G Guidi G Favaloro EJ Quality standards for sample collection in coagulation testing. Semin Thromb Hemost. 2012;38:565–75. 10.1055/s-0032-1315961 22669757

[6] Favaloro EJ (Adcock) Funk DM, Lippi G. Pre-analytical Variables in Coagulation Testing Associated With Diagnostic Errors in Hemostasis. Lab Med. 2012;43:1–10. 10.1309/LM749BQETKYPYPVM

[7] Plebani M Sciacovelli L Aita A Quality Indicators for the Total Testing Process. Clin Lab Med. 2017;37:187–205. 10.1016/j.cll.2016.09.015 28153366

[8] Lippi G Ippolito L Favaloro EJ Technical Evaluation of the Novel Preanalytical Module on Instrumentation Laboratory ACL TOP: Advancing Automation in Hemostasis Testing. J Lab Autom. 2013;18:382–90. 10.1177/2211068213491747 23736064

[9] Quick AJ Stefanini M The chemical state of the calcium reacting in the coagulation of blood. J Gen Physiol. 1948;32:191–202. 10.1085/jgp.32.2.191 18891145 PMC2147133

[10] Covance eNewsletter. Clotted Hematology and Coagulation Specimens What they are? Why they happen? What can be done? Available at: http://insite.covance.com/archives/2013/issue9/clotted_hematology_coag.pdf. Accessed February 3rd 2019.

[11] Clotted Samples in the Clinical Laboratory 2014.

[12] Arcidiacono S Callahan J Doyle M Triscott M The Utility Of Clotting Reaction Curve Error Messages On Il ACL Top Family Coagulation Analyzers: 212. Int J Lab Hematol. 2009;31:63–4.

[13] Doubleday K, Kumnick S, editors. Clot signature curves and the ACL advance. Instrumentation Laboratory. Part. No 98083-40 Rev. 1

[14] Ver Elst K Vermeiren S Schouwers S Callebaut V Thomson W Weekx S Validation of the minimal citrate tube fill volume for routine coagulation tests on ACL TOP 500 CTS®. Int J Lab Hematol. 2013;35:614–9. 10.1111/ijlh.12099 23663653

[15] Clinical and laboratory standards institute (CLSI). Collection, Transport, and Processing of Blood Specimens for Testing Plasma-Based Coagulation Assays and Molecular Hemostasis Assays; Approved Guideline - Fifth Edition. CLSI Document H21-A5. Wayne, PA:CLSI,2008

